# Pedagogical tools to explore Cartesian mind-body dualism in the classroom: philosophical arguments and neuroscience illusions

**DOI:** 10.3389/fpsyg.2015.01155

**Published:** 2015-08-10

**Authors:** Scott Hamilton, Trevor J. Hamilton

**Affiliations:** ^1^International Relations Department, London School of Economics and Political Science, London, UK; ^2^Department of Psychology, MacEwan University, Edmonton, AB, Canada; ^3^Neuroscience and Mental Health Institute, University of Alberta, Edmonton, AB, Canada

**Keywords:** Descartes, Cartesian dualism, Heidegger, rubber hand illusion, Pinocchio Illusion

## Abstract

A fundamental discussion in lower-level undergraduate neuroscience and psychology courses is Descartes’s “radical” or “mind-body” dualism. According to Descartes, our thinking mind, the *res cogitans*, is separate from the body as physical matter or substance, the *res extensa*. Since the transmission of sensory stimuli from the body to the mind is a physical capacity shared with animals, it can be confused, misled, or uncertain (e.g., bodily senses imply that ice and water are different substances). True certainty thus arises from within the mind and its capacity to *doubt* physical stimuli. Since this doubting mind is a thinking thing that is distinct from bodily stimuli, truth and certainty are reached through the doubting mind as *cogito ergo sum*, or the certainty of itself as it thinks: hence Descartes’s famous maxim, *I think, therefore I am*. However, in the last century of Western philosophy, with nervous system investigation, and with recent advances in neuroscience, the potential avenues to explore student’s understanding of the epistemology and effects of Cartesian mind-body dualism has expanded. This article further explores this expansion, highlighting pedagogical practices and tools instructors can use to enhance a psychology student’s understanding of Cartesian dualistic epistemology, in order to think more critically about its implicit assumptions and effects on learning. It does so in two ways: first, by offering instructors an alternative philosophical perspective to dualistic thinking: a mind-body *holism* that is antithetical to the assumed binaries of dualistic epistemology. Second, it supplements this philosophical argument with a practical component: simple mind-body illusions that instructors may use to demonstrate contrary epistemologies to students. Combining these short philosophical and neuroscience arguments thereby acts as a pedagogical tool to open new conceptual spaces within which learning may occur.

## Introduction

Undergraduate students typically pursue a university education with the aim of acquiring new knowledge, certainty, or truth, about the world. Recent studies have demonstrated that as students attempt to acquire knowledge about the mind and its connection to the world, they are quickly confronted with what is known as the philosophical mind-body problem, or what is commonly referred to as “substance dualism” or “Cartesian dualism” (Fahrenberg and Cheetham, 2000). Asserting a rigid ontological and epistemological difference between the immaterial mind and the material body, psychology students that fail to critically examine this Cartesian substance dualism in greater detail place a greater emphasis upon the rote memorization or surface-learning of knowledge and facts, which Ryan (1984) has demonstrated to be less effective than processes of interpretation and comprehension associated with understanding and deep-learning. Dualist epistemology, in other words, leads to weaker applications of knowledge, and to poorer grades, in psychology classrooms (Ryan, 1984; Lonka and Lindblom-Ylanne, 1996). This also runs the risk of tacitly transferring uncritical scientific dualistic beliefs into future scientific, paramedical, and medical professions (Demertzi et al., 2009). In order to explore this tacit dualism in the classroom, therefore, this article provides pedagogical tools that may be embraced by instructors and students alike: first, by offering an alternative philosophical grounding or epistemology for their learning and teaching, based not in the dualistic philosophy of René Descartes, but in the holistic philosophy of Martin Heidegger. Second, by illustrating this alternative epistemological perspective in practice, through simple neuroscience illusions such as the “Pinocchio” and “Rubber Hand” illusions that manipulate the mind’s body representation. In doing so, an instructor can make tacit dualistic assumptions held by students prior to these arguments and exercises, more explicit, so students may examine and think more critically about them. With these differing epistemologies and hands-on demonstrations, therefore, instructors expose or challenge dualistic beliefs in their classroom, and facilitate deeper learning or understanding within their students by illustrating alternative ways of conceiving how their mind and body relates to the world.

Although it is impossible to falsify a metaphysical problem that has plagued Western philosophy since the Enlightenment (and in other forms, since Plato), the purpose of this article is not to attack Cartesian dualism itself, nor is it to examine every single philosophical perspective that is contrary or alternative to dualistic thinking. This is a complex and gargantuan philosophical and scientific task that lies outside the scope of this short article. Instead, it aims to provide students and instructors with only one alternative to dualism in both thought (epistemology) and action (classroom practice), in order to catalyze a different way of thinking within the classroom: a more holistic way of thinking and understanding how the mind and body interconnect, overlap, or exist, that differs from the surface-level epistemological assumptions tacitly undergirding mind-body dualism. In doing so, it aims not to counter or disprove metaphysical dualisms, but to open new spaces for students to think critically about their world, and other philosophies and practices that might also disclose it in different ways more amenable to deep-learning and understanding. Instructors will benefit their students by promoting these critical faculties and perspectives through a deeper engagement of both science and philosophy.

## Cartesian Dualism

René Descartes (1596–1650), was a French mathematician, philosopher, and scientist of the 17th century. The founder of analytical geometry, he is now more commonly referred to as the father of modern philosophy due to his revolutionary reformulation of how truth, certainty, and the mind and body, are understood ontologically and connected epistemologically. Prior to Descartes, the composition of the mind was generally ascribed to the way the “soul” arranged sensory stimuli to form thought, as was proclaimed by Catholic orthodoxy. The mind and body were fused within a person as one whole, and truths and certainties steering the soul were determined *a priori* by God. The body operated mechanistically, akin to animal-like automata; human agency, mind, and thought, were derived from the workings of the soul as prescribed by God and articulated through Church doctrine.

Rather than simply attributing all of human thought and being to God, Descartes’s rationalism posited a revolutionary new foundation for truth and certainty: the subject’s rational and thinking mind, or the “I” of subjectivity (Descartes, 1998). This new rationality of self-certainty was grounded upon the capacity for radical skepticism, or *doubt*. Under the spell of Cartesian doubt, all empirical stimuli emanating from the material world through bodily sight, taste, touch, etc., could always mislead the mind, since, like in a dream, the mind cannot be certain these physical sensations are real. “I will suppose,” wrote Descartes, “not a supremely good God, the source of truth, but rather an evil genius, supremely powerful and clever, who has directed his entire effort at deceiving me.” (Descartes, 1998, p. 62) With no way to be certain that “the air, the earth, colors, shapes, sounds,” or any of the *res extensa* comprising our sensed body and the substantive material world actually exists, Descartes claimed that only one inexorable certainty and truth alone remained: “I see very clearly that, in order to think, it is necessary to exist” (p. 18). To doubt, is still to *think*; and to think, is to exist or to *be*. Hence, Descartes’s famous maxim that undergirds dualist epistemology to this day: “I think, therefore I am” (*cogito ergo sum*) (p. 18). Under this maxim, we can thus be certain of our own thinking minds as existing separately from our sensed substances and body, because the mind can perceive and *reason* against what our body quickly misapprehends: “what I thought I had seen with my eyes, I actually grasped solely through the faculty of judgment, which is in my mind.” (p. 68). What my body tells me are two different substances, water and ice, my mind *reasons* are the same substance. Although later followers of Cartesian rationalism and dualism abandoned Descartes’s antiquated belief that the soul (mind) could meet and impact the “vital spirits” of the mechanical workings of the body through the brain’s pineal gland, the dualistic epistemology he posited between thought/matter, subjectivity/objectivity, and mind/body, remains tacitly entrenched in Western science, philosophy, and scientific and cultural discourse, to this very day.

For instance, instructors may easily refer to recent Hollywood films such as *The Matrix* (1999) or *Inception* (2010) as cultural examples that illustrate (and risk entrenching) dualistic epistemology: like Descartes’s “evil genius,” these films stress that truths and certainties derived from sensed bodily stimuli may indeed be dreamlike or misleading, but may be corrected or overcome by a thinking self, and its rational mind. Here, the mind is portrayed as being firmly distinct, separate, and in need of liberation from, the prison of the body whose stimuli cannot be trusted. Indeed, recent studies have demonstrated that dualistic beliefs are retained throughout a student’s education, regardless of disciplinary background and training in otherwise scientific, medical, and paramedical environments. Demertzi et al. (2009) studied the presence of dualistic beliefs in a sample of students from the University of Edinburgh in Scotland and health-care workers and the general public at the University of Lèige in Belgium. The majority of undergraduate students surveyed agreed that “the mind and brain are two separate things” and slightly less than half of the participants in the Liege survey agreed to this statement (Demertzi et al., 2009). Interestingly, almost half of health care professionals surveyed also agreed with this dualistic statement. These findings highlight the continued presence of dualistic beliefs throughout society despite neuroscience studies, particularly those using functional magnetic resonance imaging (fMRI), that continue to suggest that neural activity is responsible for psychological phenomenon (Greene et al., 2001; Farrer and Frith, 2002), and therefore that the brain is the origin of the mind. However, belief in dualism is also dependent on perceived strength of the evidence provided by scientific studies. When subjects are exposed to weak neuroscientific evidence describing psychological phenomenon, they have an increased tendency to believe in the presence of a soul. Conversely, when the neuroscientific evidence is strong, subjects are more likely to decrease their belief in the soul (Preston et al., 2013). Therefore, in a neuroscience or psychology classroom it is important to provide an accurate description of current research, so that the relationship between the mind, brain, and body, may be problematised and explored more critically by students and instructors alike.

The resilience of dualistic beliefs is also indicated by studies demonstrating that some medical patients with somatoform disorders are reluctant to attribute ailments to psychological symptoms rather than to physical symptoms (Stone, 2006 *as cited in* Demertzi et al., 2009), and some prefer to try and identify physical rather than psychological causes of medically unexplained symptoms (Geist et al., 2008), reinforcing the Cartesian binary division between mind and matter. Indeed, beliefs that the “soul” survives bodily death and destruction remains prevalent within the scientific community, and dualism even affects psychology’s own “neuroscientific thinking” by implying that the material brain generates, yet remains radically separate from, the mind (Demertzi et al., 2009). But could dualistic thought patterns actually be detrimental to everyday life? Recently, researchers have used priming procedures to induce either “dualist” or “physicalist” beliefs and found that in the dualist condition subjects engaged in less healthy attitudes and behaviors (Forstmann et al., 2012).

## Using Basic Philosophical Arguments in Psychology Classrooms: Descartes and Heidegger

If training or education in a university setting still risks the retention of dualism’s presuppositions, then how might instructors encourage a more critical and meaningful analysis of dualist epistemology amongst their students? Philosophy and science can work in tandem here when it comes to examining subtle presuppositions of the mind-body problem and dualistic epistemology. To take only one example from the vast canvas of Western philosophy, let us examine the basic work of Martin Heidegger, which can act as an example of how an instructor might provide their students with a different, yet equally powerful, epistemological perspective and understanding of the world. The aim here is not to supplant nor to disprove the metaphysical presuppositions of dualism, but to indicate how an instructor might approach it using different philosophical and epistemological perspectives.

Philosophically, Cartesian dualism’s assertion of an ontological separation between mind and matter was radically undermined by the publication of Martin Heidegger’s *Being and Time*, and its revolutionary concept of “being-in-the-world” (Heidegger, [1927] 1962). Contrary to the binaries of dualistic epistemology, Heidegger argued that our modern and naturalized concepts of subjectivity, “I,” cogito, or mind, could *never* be separated or detached from objects, matter, or the world, as dualism assumes. For Descartes, when a subject sees, cognizes, and uses everyday objects such as a hammer, a doorknob, a pencil, etc., their thinking mind crosses an ontological gulf to the world of matter and the body, accruing sensory stimuli and empirical properties of these material substances so as to compute, reason, and then actuate, their rational use in the mind (Heidegger, [1927] 1962, p. 128). Heidegger’s fundamental insight, however, was that each of these “objects” makes sense to a “subject” not through any mental rationalization or detached thinking or combination of sensed properties. Instead, things have meaning or are disclosed to us as humans, only through pre-reflective, learned, and everyday contextual practices or *uses*. In other words, in modern times, an object as simple as a hammer is recognized as something that can pound a nail into wood only *after* a person has already been socialized into cultural, linguistic, and discursive practices in a shared world, that teaches them that this “hammer-thing” is used in this specific way. A stick of wood and blob of metal is, thus, disclosed to us as a hammer only after our enmeshment in a shared world discloses to us the social and cultural contexts and circumstances that make it intelligible as something *to* use. “Such an entity can “meet up with” Dasein [i.e., the “thinking” person] only so far as it can, of its own accord, show itself within a *world*” (Heidegger, [1927] 1962, p. 84). The point here is that an instructor can use Heidegger’s philosophy as a tool, to show how a student’s implicit “being in the world” includes and undergirds the metaphysical presumptions of dualism they once took for granted or assumed.

Upon deeper inspection, when a person is in the actual process of hammering a nail, turning a doorknob so as to walk through a doorway, using a pencil to write down psychology notes, etc., the ontological separation sustaining Cartesian mind-body dualism breaks down. Why? According to Heidegger, each of these “objects” can be made intelligible and understood because they are enmeshed within countless and enormously complex and interdependent historical, social, and cultural networked contexts, that combine to give an “object” its naturalized meaning, significance, and use. Although we contextualize a hammer as an object to pound nails, an ancient Greek, or an alien from another planet, would lack the social, cultural, and psychological contexts that makes this “thing” meaningful or intelligible to us: boards, nails, saws, screws, structures, bookshelves, ladders, paints, etc., combine to form an “equipmental totality” that is historically and culturally unique to us, yet is acquired and made implicit through our everyday socialized use (Heidegger, [1927] 1962). These are the countless and networked things we know are related and associated with *every thing* that our world makes intelligible: its background associations and contexts that make it an intelligible thing, to use in a particular way. Understood in this light, no amount of rational thinking can *ever* inform us what something as simple as a hammer actually *is* in a dualistic context. A student could never look at a hammer, for the first time, and simply rationalize its use. Instead, “What makes agency possible is not some underlying [material] substrate, not some mental substance, but is rather the way our life stories unfold against the backdrop of practices of a shared, meaningful world.” (Guignon, 2006, p. 9) Hence, Heidegger’s famous dictum “being-in-the-world” eliminates the supposed gulf or dualism between subject and object, mind and body, etc (Heidegger, [1927] 1962). As human beings, upon birth, we are “thrown” into a particular world that imprints within us particular and pre-reflective ways of understanding, communicating, and navigating, practices and our own “being” in a world of intertwined contexts and meanings. Again, the point here is that the mind and body are not inherently different substances, until modern, everyday, and tacit cultural practices, such as the Cartesian dualist epistemologies examined above, reveals or discloses them to us in our own particular historical contexts, as two separate entities. In order to use this philosophy as a pedagogical tool in their classroom, an educator in an introductory psychology class may therefore lecture on the Heideggerian examples given above, then use classroom discussion or a group activity to determine whether students understand the concept and this contrary epistemological perspective. For example, “Name an example of a recent movie or television series that assumes Cartesian dualism, and counter this with Heidegger’s [or, another philosopher of the instructor’s choice] position.” See Table 1 for some simple examples. As will be examined below, therefore, psychological illusions that problematize commonsensical (mis)understandings of our body’s existence in space will help to illustrate how our body’s ongoing situatedness in a world is often forgotten or taken for granted.

**TABLE 1:** **A classroom exercise to discuss and critique dualistic themes in the media**.

**Media**	**Dualistic theme**	**What might Heidegger say? What is the alternative philosophic/epistemological perspective?**
Example: The Matrix (1999)	The mind is a separate entity from the body, and can’t trust its senses; it can control them! The “self” is ultimate, *choosing* its destiny and shaping its world.	The contextual “world” within the Matrix, within which Neo was raised, would actually make the “real” world completely different and incommensurable to him when he was eventually exposed to it. So, unlike the movie’s claims, the Matrix and the “real world” would actually have to be *very* similar for the mind’s ontological/epistemological perspectives of being to share and to make sense of both worlds, simultaneously. It’s clear the separation between mind/body or mind/world posited by the movie couldn’t be possible.
Ghost (1990) Being John Malkovich(1999) Other Movies:		

## Body Representation and Body Schema

In order for any physical interactions between an individual and the external world to occur, such as hammering a nail with a hammer, using a pencil in a classroom, or avoiding a pylon while walking down the street, the mind must have a concept of the body’s position in space. The mind must be situated in-a-world. Proprioceptors are receptors located in muscles and joints that relay information about muscle stretch and joint angle to the thalamus and eventually the somatosensory area of the cerebral cortex. The mind’s schema of the body incorporates proprioceptive cues with other senses, namely vision and feedback from the motor system that allow humans (and likely most other animals) to mentally model where the body is in its external space. The body schema plays a role in the constant production of awareness of body configuration by associating various perceptual inputs, calculating and reconstructing any missing information, and detecting and resolving conflicts (Graziano and Botvinick, 2002). In the classroom setting it can be a difficult task to clearly demonstrate that the mind and the body may be one and the same. However, there are some simple perceptual demonstrations that can be used for this purpose that alter the mind’s body schema. Thus, by altering input to the body’s sensory system the mind can become confused and produce illusory conclusions about what is happening to the body. If subjective experiences are brought about by the mind, which exists differently than our sensed substances and body, then would illusions caused by an alteration of sensory perception have an effect on the mind? According to interaction dualists, like Descartes, the mind and body are causally linked and can communicate with one another and this interaction between the soul and body occurs through the pineal gland. However, if the mind is generated by the activity of neuronal circuitry in the brain that is partially influenced by the sensory systems of the body (whose connections are independent of the pineal gland), then confusing input to the brain may result in altered, illusory, perception of the mind. In fact, this notion is how some scientists have developed techniques to treat “phantom limb pain.” In this condition, amputees still feel pain in their limb that has been removed. It is recommended that educators teach this fascinating condition to students and discuss the “mirror-box” as treatment for this condition (McGeoch and Ramachandran, 2012; also see Youtube link^1^). Following is a description of two simple, yet thought-provoking, illusions that can be performed in a classroom. The use of these demonstrations can be a salient mechanism to maintain the focus of the students and use a different modality to discuss mind-body duality. These perceptual illusions described below can alter the mind’s representation of the body in space.

## Pinocchio Illusion

**FIGURE 1:**
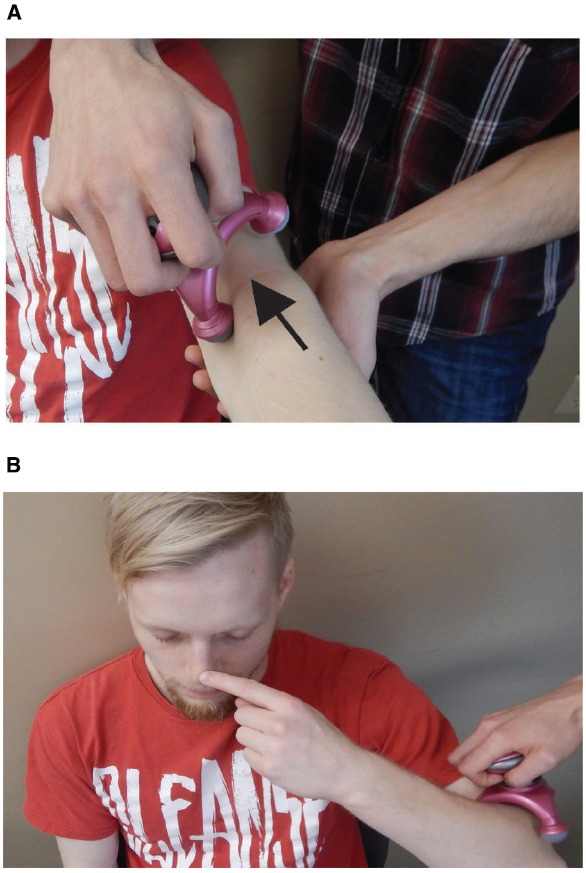
**The pinocchio illusion. (A)** The participant extends their dominant arm so that vibration may be applied to biceps tendon. It is important to first place the vibration on the crux of the arm (arrow). **(B)** The subject then bends their arm, closes their eyes, and places their index finger on their nose. Usually after 1–2 min the subject will feel that their nose is growing.

In the 1940s classic Walt Disney movie, *Pinocchio* was a fictional marionette character that was made of wood, and is best known because his nose grew whenever he told a lie. By vibrating the biceps brachii tendon, that sends proprioceptive input to the brain, it is possible to evoke the feeling of the nose growing and thus has been coined *The Pinocchio Illusion* (Lackner, 1988). This simple illusion can be produced by having a subject close their eyes and touch their nose with a finger while the biceps tendon of that arm is vibrated (see Figure 1). The phantom sensation that is produced in some participants (Burrack and Brugger, 2005) is an elongation of the nose. The vibration of the biceps tendon triggers muscle spindes to send proprioceptive input to the brain that signals the extension of the arm (an increase in elbow joint angle; DiZio and Lackner, 2002). Because the brain is also getting tactile input from both the nose and finger tip that they are touching, the combination of these stimuli are combined in the brain to conclude, incorrectly, that the nose is growing/moving away from the face. Note that the dominant arm should be used along with a vibration frequency of about 100 Hz for an optimal effect (Burrack and Brugger, 2005) and therefore, basic hand massagers may not initiate the illusion.

## Rubber Hand Illusion

**FIGURE 2:**
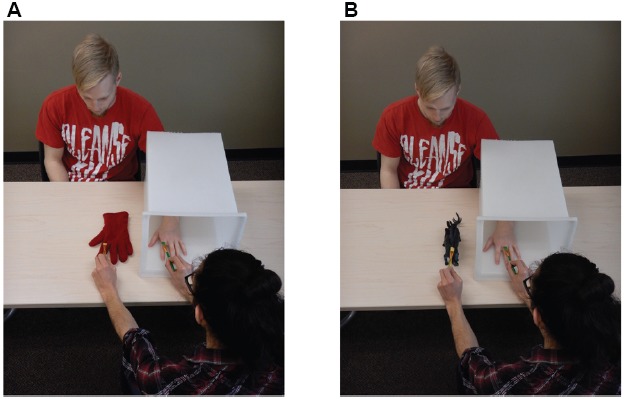
**The rubber hand illusion. (A)** The participant extends their left hand into the enclosure so they cannot see their hand. They stare at the rubber hand, or glove (shown here), that is in an identical orientation to their hand in the enclosure. The person administering the illusion uses both paint brushes to touch the participant and the rubber hand in an identical manner. The illusion usually takes 1–2 min to take effect. **(B)** In some participants who are very susceptible to the illusion, another object may be placed where the rubber hand was. In this example, the person administering the illusion would “paint” the dinosaur and the hand in an identical manner. For some people the dinosaur will feel like it is a part of the body.

Another illusion, which it is commonly called “the rubber hand illusion” (Botvinick and Cohen, 1998), tricks the mind into *feeling* that an external object (commonly a rubber hand) is part of the body (see Figure 2, also see^2^). In this illusion the participant directs their vision to a rubber hand on a table while their corresponding left or right hand is placed out of view. The person administering the illusion then uses a paintbrush to touch the rubber hand in an identical fashion to the real hand. After a few minutes of “painting” the fingers, knuckles, and hand most participants will feel like the rubber hand is part of their own body. This is due to the conflicting input from the external stimuli received by the photoreceptors in the eyes and mechanoreceptors and proprioceptors in the skin. This conflicting input travels from the thalamus to the somatosensory cortex, then an association area in the cortex where the brain makes the ultimate decision, which is incorrect, that the object located outside of the body, must be part of the body. In this sense, the brain has altered its mental image of the body schema to incorporate the rubber hand. Researchers have demonstrated that this is an alteration of the body’s perception of where the hand is located in space by asking participants to complete a follow-up test. After the rubber hand illusion was administered to the left hand, participants were instructed to close their eyes and line up the right hand (underneath the table) to where they believed their left hand was located. They found that there was a significant shift in where the participants thought their left hand was in the direction of the illusory rubber hand, and the strength of this distortion was correlated with the efficacy of the rubber hand illusion itself (Botvinick and Cohen, 1998). Some studies report using a paint brush and model of a human hand (Botvinick and Cohen, 1998), however, a glove and tactile stimulation of the hand with fingertips can also be used if a model of a rubber hand and paintbrushes are not available.

## Summary

This perspectives article has outlined how instructors can explore the epistemological assumptions undergirding Cartesian dualism on both philosophical and scientific grounds. The philosophy of Heidegger highlights how our ongoing enmeshment and involvement in a social and cultural world makes certain objects intelligible, thinkable, and meaningful to us—such as the implicit understanding required in using something as simple as a hammer, which derives not from detached rationalizations of bodily sensory data or stimuli, but from cultural practices making its use intelligible and normal. The psychological Rubber Band and Pinocchio Illusions reinforce the basic insight that the body and mind are enmeshed in a shared world, by transgressing the supposed dualisms of mind-body highlighted by Descartes, yet with an understanding that the rational mind is not a superior *a priori* modicum for understanding truth. Even philosophy, metaphysics, and science, is undergirded by a shared sense of world that cannot emanate from a rational mind in-itself. For instance, by stimulating the body with touch or vibration, the sensory input is incorporated into the brain’s mental schema of where the body is in external space, and with the illusions described here; and this is incorrectly interpreted by the mind as an illusory conclusion (that the nose is growing, or the rubber hand is part of one’s body). The mind’s rational assumptions about its body, self, and world, are problematised. In so doing, these simple illusions can be presented alone or together with differing philosophical perspectives as pedagogical tools to educate students by promoting increased and critical thinking about dualistic presuppositions.

### Conflict of Interest Statement

The authors declare that the research was conducted in the absence of any commercial or financial relationships that could be construed as a potential conflict of interest.

## Footnotes

1 http://www.ted.com/talks/vilayanur_ramachandran_on_your_mind?language=en

2 https://www.youtube.com/watch?v=TCQbygjG0RU
